# The use of tibial Less Invasive Stabilization System (LISS) plate [AO-ASIF] for the treatment of paediatric supracondylar fracture of femur: a case report

**DOI:** 10.1186/1749-799X-5-10

**Published:** 2010-02-18

**Authors:** Hoi Yan Lam, Chun Kwong Lo, Kai Yin Cheung

**Affiliations:** 1 Department of Orthopaedics and Traumatology, Alice Ho Miu Ling Nethersole Hospital, 11 Chuen On Road, Tai Po, New Territories, Hong Kong

## Abstract

Paediatric supracondylar fractures of the femur are not common. The treatment options depend on the age of child, the site of the fracture, the pattern of injury and the surgeon's preference. We report a case of an 11-year old boy who sustained a comminuted displaced supracondylar fracture of the femur and was treated with indirect reduction and internal fixation with the Less Invasive Stabilization System (LISS) tibial plate.

## Background

Paediatric supracondylar fractures of the femur are uncommon. There are different modalities of treatment depending on the age of child, the site and the fracture pattern. The use of traction, hip spicas in young children, external fixators, flexible intramedullary nails or even plating had been reported but each had its own limitations. The Less Invasive Stabilization System (LISS, Synthes) combines minimally invasive internal fixation with fixed-angle screws. To our knowledge, there has been no report about fixation of paediatric distal femur fractures with a LISS tibial plate. We report a case of an 11-year old boy who suffered from a displaced comminuted supracondylar fracture of the femur and was treated with close reduction and internal fixation with a LISS tibial plate.

## Case Presentation

**Figure 1:**
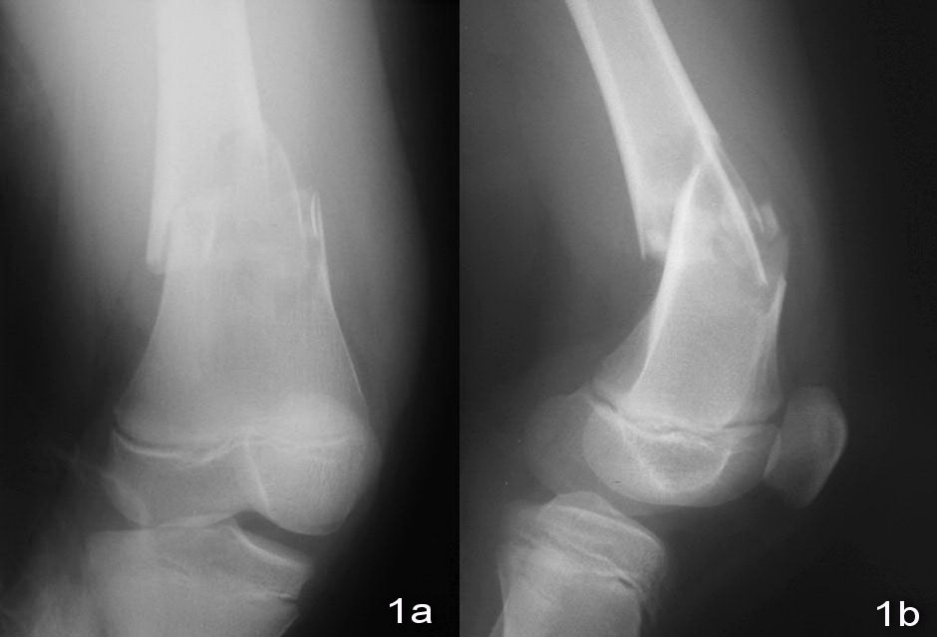
**X-ray of right knee showed comminuted supracondylar fracture of femur**.

**Figure 2:**
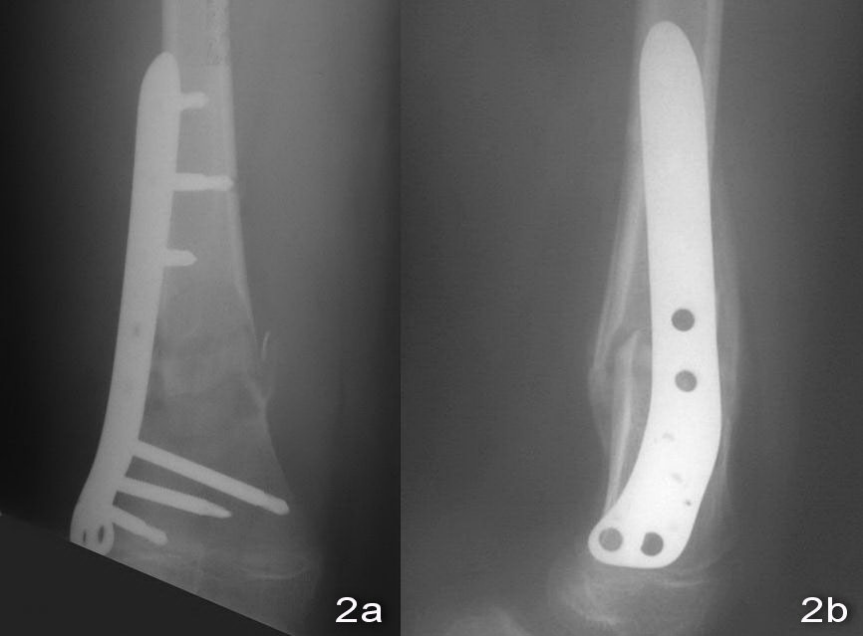
**Post operative X-ray of right knee showed good alignment**.

**Figure 3:**
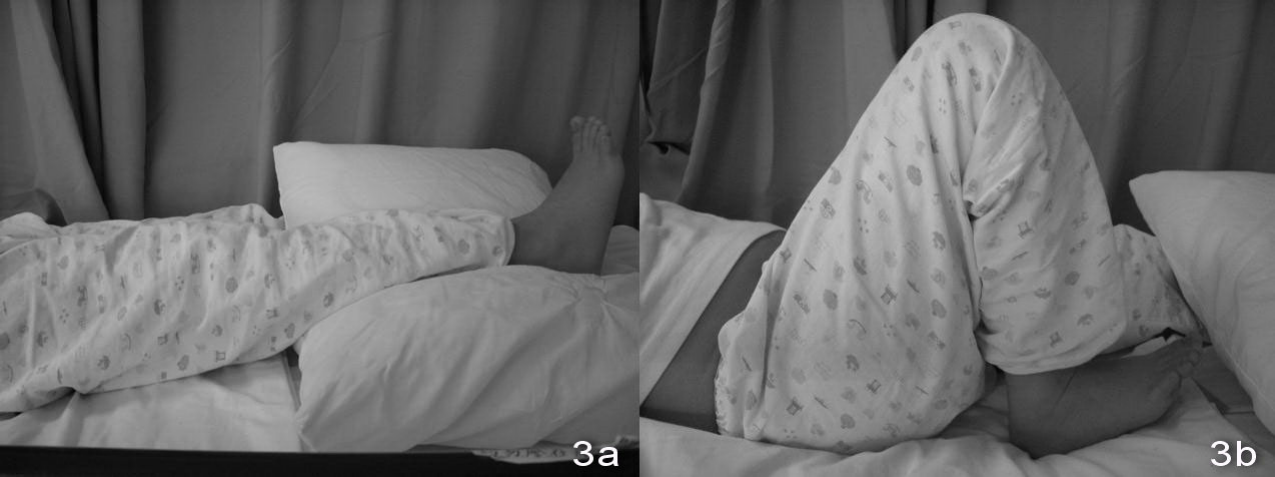
**Patient could achieve full range of motion after operation**.

**Figure 4:**
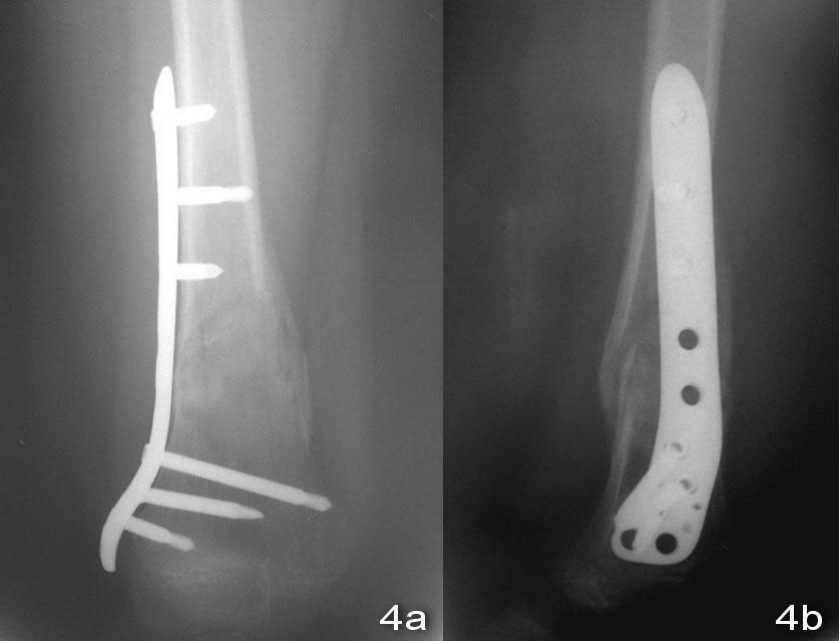
**X-ray of right knee showed united fracture**.

**Figure 5:**
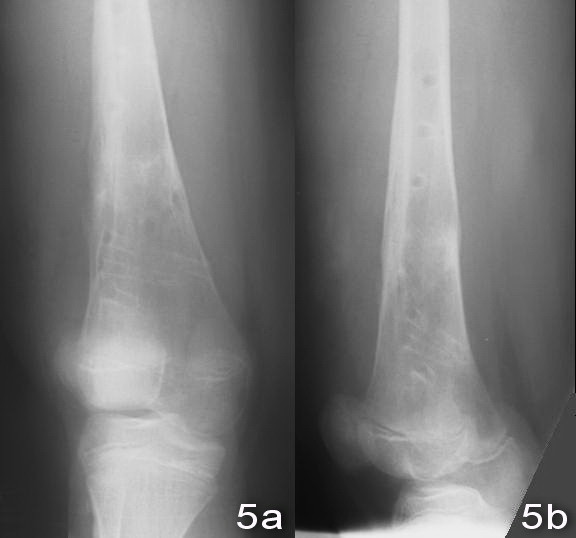
**X-ray of right knee after implant removal**.

An 11-year old boy sustained a fall during a soccer game. He landed on his right knee and complained of severe right leg pain and swelling after the injury. There was no associated injury. Physical examination showed deformity with swelling over the right distal thigh. There was tenderness over the distal femur. There was no neurovascular deficit. X-ray of the right knee showed a displaced supracondylar fracture of the right distal femur with comminution both the medial and the lateral cortex. The fracture was classified as AO/ASIF (Arbeitsgemeinschaft Fur Osteosynthesefragen/Association for the Study of Internal Fixation) Type 33A [Figure 1a and 1b]. Closed reduction and fixation with tibia LISS plate was performed. (The reasons for choosing the LISS tibial plate were illustrated in the Discussion Section.) We performed lateral approach with incision over the right distal femur. After closed reduction of the fracture with satisfactory alignment, we inserted the tibial LISS plate in submuscular plane and temporarily fixated it with Kirschner wires. We then inserted the locking screws through the jag. Intra-operatively, we took a bone biopsy to exclude the possibility of a pathological fracture and it showed no malignant cells. Post-operatively, he was on non-weight bearing walking for six weeks, partial-weight bearing walking for another six weeks and was given early knee mobilization exercises [Figure 2a and 2b]. On two months post-operatively period, there was no knee pain and the range of motion of the right knee was full [Figure 3a and 3b]. X-ray of the right knee showed that the fracture was united [Figure 4a and 4b]. He had implant removal one year after the operation [Figure 5a and 5b]. On post operative period two years, the right knee range of motion was full (0-130 degrees) and there was no right knee pain.

## Discussion

Supracondylar fractures of the femur are uncommon. In children, they may be associated with musculoskeletal conditions, such as spinal muscular atrophy, osteogenesis imperfecta. Smith et al found that the incidence of supracondylar fractures was 12% of all femoral fractures and 7 out of the 12 supracondylar fractures in their study of 112 femoral fractures were displaced and 3 were not due to bone insufficiency [1].

Undisplaced supracondylar fractures of the femur can be easily managed by closed means with a molded long leg plaster cast. However, there is no ideal method in the literature for the management of displaced supracondylar fracture. Displacement makes the fracture unstable and management can be difficult so that operative intervention is more likely. There is no data concerning the effect of the fracture on leg length and the ability of the femur to deformity remodeling at this level.

The treatment of displaced supracondylar fractures of the femur depends on the age and size of the child, the site, the pattern of the fracture and its associated injury. When treating the displaced supracondylar fracture, the traditional method of traction may fail due to the unbalanced pull of the gastrocnemius or adductor muscle causing difficulty in controlling the alignment. Prolonged bed rest and hospital stays have negative social and psychological effects on growing children. The use of hip spica casting is difficult in older children and the control of alignment cannot be guaranteed.

Butch et al advocated closed reduction and percutaneous cross pin fixation via epicondyles with smooth Kirschner wires or Steinmann pins, depending on the size of the femur, similar to the treatment of a supracondylar fracture of the humerus [2]. However, this method may need post-operative cast immobilization and there is a chance of intra-articular pin placement, causing septic arthritis and a risk of damaging the growth plate.

An external fixator is also used for the treatment of paediatric supracondylar fractures but there may be problems with pin tract infection and growth plate disturbance due to intraepiphyseal placement of half pins especially in fractures with relatively short metaphyseal fragments. Moreover, there are cosmetic concerns with pin scarring and the chance of refractures after removal of external fixators. Sabharwal et al. used an Ilizarov external fixator to try to avoid intraepiphyseal placement of pins and as the Ilizarov device appears more modular, it can allow multiplaner pin fixation and better control of alignment [3]. It is good for patients with open fractures or very comminuted fractures but the Ilizarov device is not comfortable to the children.

For adolescents with closed femoral physis, we would consider locked intramedullary nails. It is important to have enough space for two locking bolts in the distal fragment. However, this cannot be used in growing children. The design of flexible intramedullay nails, either steel or titanium, introduced percutaneously may avoid the violation of growth plates. However, it may be difficult to insert and control the alignment in distal comminuted fractures. At the same time, the nail may back out causing skin irritation.

Recently, Kanlic et al used the principle of bridging plate with Low Contact Dynamic Compression Plates (LC-DCP) for fracture fixation [4]. This allows more anatomical and stable fixation. With the technique of indirect reduction for secondary bone healing, the LC-DCP can be inserted over the submuscular plane and it can decrease soft tissue dissection and preserve the bone fragment blood supply. Though the conventional plating provides excellent stability and maintenance of length and alignment but it is at the cost of increase the soft tissue injury at the fracture site and increases the chance of femoral overgrowth [4]. For comminuted fractures with short distal fragments, it may be better to use fixed-angle devices (like dynamic compression screws or condylar blade plates) for better alignment control especially to prevent varus displacement. However, we need to avoid the growth plate during the insertion of dynamic compression screws or the condylar blade. The LISS was developed for osteoporotic or comminuted fractures of the distal femur. It has threaded screw heads which lock into threads in the plate to create a screw-plate construct and act as a fixed-angle device. It can place up to six locked screws in the distal fragment. It had the advantages in the setting of osteoporotic bone, articular fractures and extremely short distal fragments [5].

In our case, the physis of the distal femur was not closed yet and the supracondylar fracture was distal. At the same time, there was comminution over both the medial and lateral cortex. Moreover, the fracture was displaced and the boy was quite big for his age. The option of percutaneous Kirschner wires and flexible intramedullary nails was not a good choice as it is difficult to control the alignment when there was comminution. With the limitation of small distal fracture fragments and a fracture site close to the physis, it was difficult to insert a dynamic condylar screw, condylar blade plate or Ilizorav external fixation. The use of a dynamic compression plate was also unsuitable as it was not strong enough to control the distal fragment. Since there was also no medial support provided by a dynamic compression plate, varus deformity might occur due to the medial comminution. We had tried to template the usual distal femur LISS plate but the size was too large for the child's femur. For the LISS tibial plate, the size was quite a good fit and was well-contoured over the distal femoral condyle and the multiple distal locking screws had better control and fixation of the fracture fragment. Moreover, we could avoid the disturbance of distal femoral physis by the use of this implant. During the insertion of the LISS tibial plate, we needed to have a good template and plan especially for the insertion of locking screws over the distal femur for the best purchase of bone while avoiding violation of the growth plate. The disadvantages were that the patient might need another operation for implant removal and the implants were expensive compared to traditional plates.

## Conclusion

In the literature, there is no report of the use of LISS tibial plates for the treatment of paediatric supracondylar fractures of the femur. They may be considered for use in paediatric femur fractures with osteopenia, comminution and extremely short distal fragment in adolescents with open physis.

## Consent

Written informed consent was obtained from the patient for publication of this case report and any accompanying images. A copy of the written consent is available for review by the Editor-in-Chief of this journal

## Conflict of interest statement

I declare that I have no competing interests in receiving reimbursements, fees, funding or salary from an organization, not holding any stocks or shares in an organization that may in any way gain or lose financially from the publication of this manuscript, either now or in the future. HYL, CKL, KYC

I declare that I do not hold or currently applying for any patents relating to the content of the manuscript or receive reimbursements, fees, funding or salary from an organization that holds or has applied for patents relating to the content of the manuscript. HYL, CKL, KYC

I declare that I have no other financial or non-financial competing interest in relation to this paper. HYL, CKL, KYC

I declare that I have not received reimbursements, fees, funding or salary in the past five years from any organization that may in any way gain or lose financially from the publication of this manuscript either now or in the future. HYL, CKL, KYC

## Authors' contributions

HYL is responsible for literature review and writing the manuscript, CKL and KYC are responsible for the idea of the method of fracture fixation, operation of this patient and reviewing the manuscript.

HYL, CKL and KYC have read and approved the final manuscript.
